# Mediation of the association between disadvantaged neighborhoods and cortical microstructure by body mass index

**DOI:** 10.1038/s43856-023-00350-5

**Published:** 2023-09-15

**Authors:** Lisa A. Kilpatrick, Keying Zhang, Tien S. Dong, Gilbert C. Gee, Hiram Beltran-Sanchez, May Wang, Jennifer S. Labus, Bruce D. Naliboff, Emeran A. Mayer, Arpana Gupta

**Affiliations:** 1 Vatche and Tamar Manoukian Division of Digestive Diseases, David Geffen School of Medicine, University of California, Los Angeles, CA USA; 2 Goodman-Luskin Microbiome Center, University of California, Los Angeles, CA USA; 3 G. Oppenheimer Center for Neurobiology of Stress and Resilience, University of California, Los Angeles, CA USA; 4 Division of Gastroenterology, Hepatology and Parenteral Nutrition, VA Greater Los Angeles Healthcare System, Los Angeles, CA USA; 5 Department of Community Health Sciences, Fielding School of Public Health, University of California, Los Angeles, CA USA; 6 California Center for Population Research, University of California, Los Angeles, CA USA

**Keywords:** Obesity, Stress and resilience

## Abstract

**Background:**

Living in a disadvantaged neighborhood is associated with worse health outcomes, including brain health, yet the underlying biological mechanisms are incompletely understood. We investigated the relationship between neighborhood disadvantage and cortical microstructure, assessed as the T1-weighted/T2-weighted ratio (T1w/T2w) on magnetic resonance imaging, and the potential mediating roles of body mass index (BMI) and stress, as well as the relationship between trans-fatty acid intake and cortical microstructure.

**Methods:**

Participants comprised 92 adults (27 men; 65 women) who underwent neuroimaging and provided residential address information. Neighborhood disadvantage was assessed as the 2020 California State area deprivation index (ADI). The T1w/T2w ratio was calculated at four cortical ribbon levels (deep, lower-middle, upper-middle, and superficial). Perceived stress and BMI were assessed as potential mediating factors. Dietary data was collected in 81 participants.

**Results:**

Here, we show that worse ADI is positively correlated with BMI (*r* = 0.27, *p* = .01) and perceived stress (*r* = 0.22, *p* = .04); decreased T1w/T2w ratio in middle/deep cortex in supramarginal, temporal, and primary motor regions (*p* < .001); and increased T1w/T2w ratio in superficial cortex in medial prefrontal and cingulate regions (*p* < .001). Increased BMI partially mediates the relationship between worse ADI and observed T1w/T2w ratio increases (*p* = .02). Further, trans-fatty acid intake (high in fried fast foods and obesogenic) is correlated with these T1w/T2w ratio increases (*p* = .03).

**Conclusions:**

Obesogenic aspects of neighborhood disadvantage, including poor dietary quality, may disrupt information processing flexibility in regions involved in reward, emotion regulation, and cognition. These data further suggest ramifications of living in a disadvantaged neighborhood on brain health.

## Plain language summary

Neighborhood disadvantage (a combination of low average income, more people leaving education earlier, crowding, lack of complete plumbing, etc.) is known to impact the health of people’s brains. We evaluated whether neighborhood disadvantage was associated with differences in the structure of people’s brains, and whether any differences were related to an unhealthily high weight and a high intake of trans-fatty acids, a component of fried fast food, on the structure of people’s brains. Based on our results, regions of the brain that are involved in reward, emotion and gaining knowledge and understanding might be affected by aspects of neighborhood disadvantage that contribute to obesity, such as poor dietary quality. This suggests that it might be important to make healthier food more readily available in disadvantaged neighborhoods to improve the health of people’s brains.

## Abstract (web summary)

Kilpatrick et al. investigate the relationship between neighborhood disadvantage, trans-fatty acid intake, body mass index, stress and cortical microstructure. Results suggest obesogenic aspects of neighborhood disadvantage could disrupt information processing flexibility in regions involved in reward, emotion regulation and cognition.

## Introduction

Living in a disadvantaged neighborhood (area deprivation) is linked to worse health outcomes, including poor brain health^1^. For example, living in a disadvantaged neighborhood is associated with decreased brain volume^2^. The key mechanisms that underlie the link between neighborhood conditions and brain health remain unclear, but obesity is one possible pathway. Individuals living in disadvantaged neighborhoods are at higher risk of obesity due to the poor quality of available foods and environments that hamper physical activity^3–5^. In particular, neighborhood disadvantage is associated with an increased intake of calories from trans-fatty acids (TFAs) and sodium^6^. TFAs (high in fried fast food) are known to contribute to obesity, especially abdominal obesity^7,8^. Additionally, chronic neighborhood stressors, which increase allostatic load^9^, can impact eating behaviors, increasing desire for highly palatable, but unhealthy foods, as a coping response^10,11^. Numerous neuroimaging studies have demonstrated that stress can alter brain structure and function, leading to food cravings, contributing to an increased risk for obesity^12–14^. Further, high body mass index (BMI) has been shown to mediate the impact of living in a disadvantaged neighborhood on reduced brain volume, suggesting its importance in the negative impact of neighborhood disadvantage on brain health^15^.

Neighborhood disadvantage, high BMI, and chronic stress have also been shown to impact the cortical microstructure as assessed by the T1-weighted/T2-weighted (T1w/T2w) ratio and myelin content^16–19^. Intracortical myelination, which refers to the myelination of axons in the cortical gray matter, affects the timing and integration of signals from multiple axons, and is critical for neural synchrony and fine-tuning of cortical circuits, affecting cognitive functioning^20–22^. The T1w/T2w ratio has been suggested as a proxy for intracortical myelination^23,24^, as it is sensitive to intracortical myelin content (sensitively of 75% for cortical demyelination in multiple sclerosis^25^); however, it shows limited specificity^25,26^ and may also reflect neurite or synaptic density^27,28^. Despite uncertainty in the biological underpinning of the T1w/T2w ratio, numerous studies suggest it has utility and biological meaningfulness in terms of cortical maturation patterns and cognition^22,29–31^.

Cortical layers vary in terms of cell populations, specific inputs and outputs, and information processing functions (e.g., subcortical vs. intercortical input, feedback vs. forward processes)^32^. For instance, the upper cortical layers (layers 1-3) contain a large fraction of myelinated inhibitory parvalbumin-positive basket cells, which play an important role in gamma network oscillations that support a variety of cognitive processes^33^, while myelinated non-gamma-aminobutyric neurons predominate in deeper cortical layers, integrating thousands of synaptic inputs^34–36^. Accordingly, examining the microstructure at different cortical layers can inform how alterations in cell populations, processes, and communication routes may be affected by adverse or stressful environments, such as living in a disadvantaged neighborhood.

**Fig. 1:**
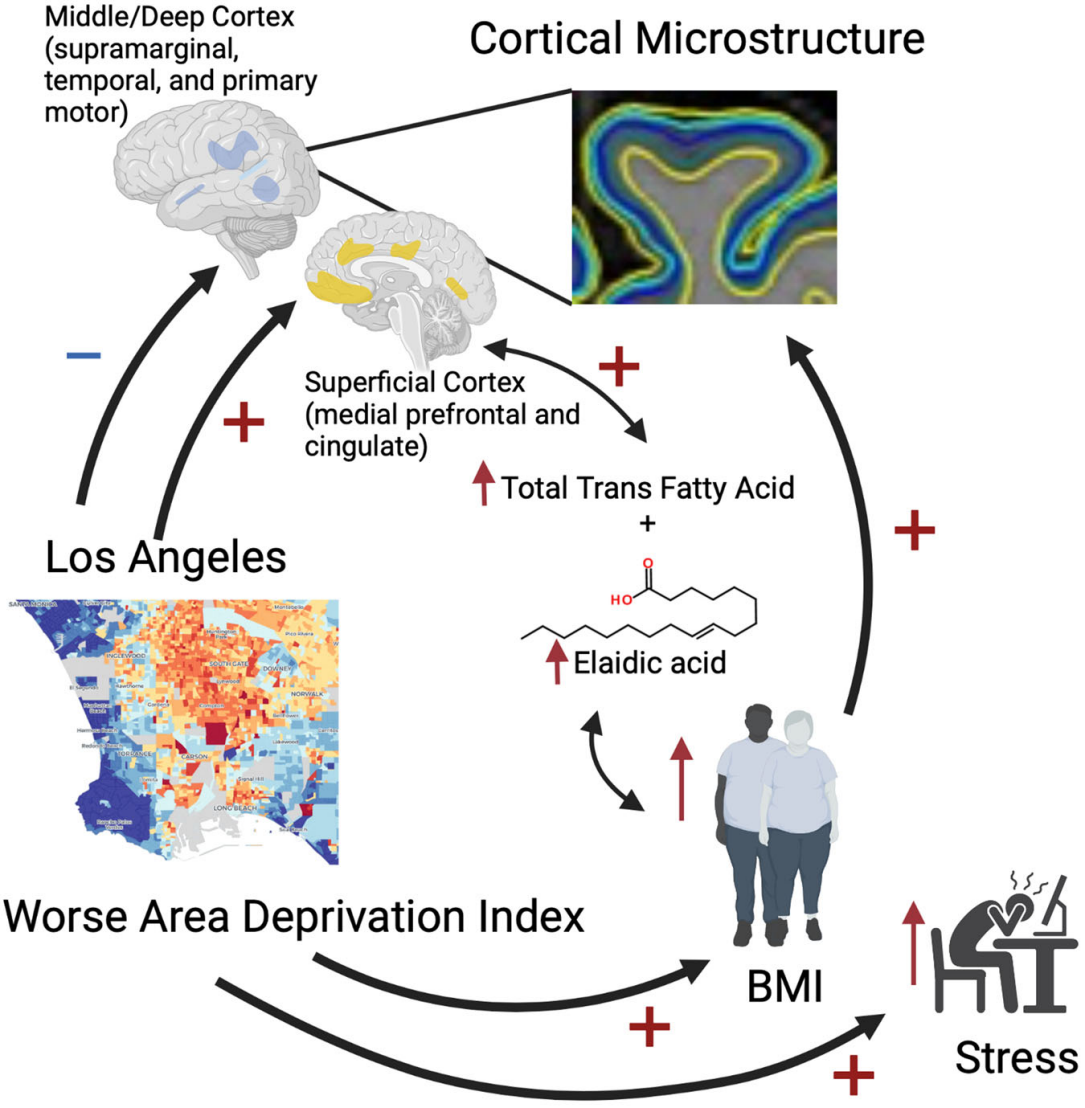
Study overview. A summary of the study is depicted. Stock images from biorender were used with permission. BMI body mass index.

We, therefore, investigated the relationship between the area deprivation index (ADI) and cortical microstructure, as assessed by the T1w/T2w ratio, at multiple cortical levels, as well as the role of potential mediators, including BMI and stress (Fig. 1). In addition, we investigated the relationship between TFA intake and the T1w/T2w ratio in a subset of participants with dietary data. We hypothesized that worse ADI would be associated with higher BMI and an obesogenic diet (characterized by high TFA intake), and higher stress levels, with negative effects on the cortical microstructure in reward-related, emotion regulation, and cognitive regions. Consistent with this, we found that worse ADI was associated with higher BMI, TFA intake, and perceived stress. We also found that worse ADI was associated with decreased T1w/T2w ratio in middle/deep cortical levels in supramarginal, middle temporal, and primary motor regions, and increased T1w/T2w ratio in middle/superficial cortical levels in medial prefrontal and cingulate regions; the latter of which was partially mediated by increased BMI and positively correlated with TFA intake.

## Methods

### Participants

Participants comprised 92 adults (27 men; 65 women) from the Los Angeles area who completed a neuroimaging session between October 30, 2019 and July 14, 2022 that included both T1w and T2w scanning and provided residential address information. Participants were recruited by flyers posted on the University of California, Los Angeles (UCLA) campus and doctor offices and handed out at community/church events in the Los Angeles area, as well as by mass emails to the UCLA community and listings on social media and clinicaltrials.gov (NCT05120908). Exclusion criteria were as follows: major neurological condition, current or past psychiatric illness, vascular disease, weight loss/abdominal surgery, substance use disorder, use of medications that interfere with the central nervous system, pregnant or breastfeeding, strenuous exercise regimen (>8 h/week of continuous exercise), weight >400 pounds, or metal implants. In addition, individuals with poor-quality images were excluded. Image quality was evaluated with the MRI Quality Control tool (MRIQC)^37^, using published thresholds for quality index 1, which reflects the proportion of voxels with intensity corrupted by artifacts normalized by the number of voxels in the background^38^. None of the enrolled participants were deemed to have poor-quality images.

The present study used data from studies approved by the Institutional Review Board (IRB) at the University of California, Los Angeles’s Office of Protection for Research Subjects (Nos. 16-000281, 20-000540, 20-000515, 20-002326 [NCT05120908]). All participants provided written informed consent. IRB approval specific to the present study was not required as de-identified data under approval were used. This study does not report on outcomes from the clinical trial, NCT05120908, as its research focus differed from that of the clinical trial. Further, the indicated inclusion and exclusion criteria pertain to the present study and are not limited to those for the clinical trial, NCT05120908.

### Assessments

Basic demographic data, as well as weight and height, were collected. BMI was calculated as weight divided by the square of the height (kg/m^2^). ADI was originally developed by the Health Resources and Services Administration several decades ago and is updated periodically. We used the 2020 ADI in the Neighborhood Atlas®^39^, based on the residential address provided by the participant. In this atlas, a neighborhood is defined as a census block group. For each census block group, the ADI is estimated by 17 neighborhood-level measures reflecting income, education, employment, and housing quality; census block groups are then ranked in terms of ADI within each state (as deciles; scores range 1–10) and nationally (as percentiles; scores range 1–100), with higher values indicating greater deprivation/disadvantage. As all participants resided within California, resulting in a non-normal distribution for the national ADI, we employed the California State ADI as the measure of ADI.

Participants also completed the Perceived Stress Scale (PSS), which is a 10-item questionnaire that assesses feelings of stress during the prior month^40^. Scores range from 0 to 40, with higher scores indicating greater stress. A review of the psychometric properties of the PSS found Cronbach’s alphas ranging .74–.89, indicating acceptable to good reliability^41^.

Diet information was collected using the web-based VioScreen Graphical Food Frequency System (Viocare Technologies, Inc., Princeton, NJ), and was available in a subset of participants (*N* = 81) as 11 participants did not provide this information. Using pictures of foods, the VioScreen Graphical Food Frequency System collects data on the use of 156 food items/groups, along with portion sizes, for the prior 3 months. For foods that are consumed at least once per month, the respondent is further queried to determine formulation or preparation method, as appropriate, and refine nutrient estimates (e.g. added sugar, fatty acid intake), which are automatically calculated. The VioScreen Graphical Food Frequency System shows similar measurement properties to those of traditional paper-based food frequency questionnaires^42^. We focused on the intake of individual TFAs reported by the VioScreen (trans-hexadecenoic acid, trans-octadecenoic acid [elaidic acid], and trans-octadecadienoic acid [linolelaidic acid]), as well as the total TFA intake. TFA intake was assessed as a component of a poor-quality diet known to contribute to obesity, especially abdominal obesity, and have harmful effects on the brain.

### Imaging acquisition and preprocessing

T1w and T2w structural images were obtained in accordance with Human Connectome Project (HCP) protocols (version 4.3) using a 3.0 T Siemens Prisma MRI scanner (Siemens, Erlangen, Germany). Spin echo fieldmaps were also acquired in anterior-posterior and posterior-anterior directions for distortion correction. The acquisition parameters for high-resolution T1w images were as follows: echo time, 1.81 ms; repetition time, 2500 ms; slice thickness, 0.8 mm; number of slices, 208; voxel matrix, 320 × 300; and voxel size, 1.0 × 1.0 × 0.8 mm. The parameters for the T2w images were as follows: echo time, 564 ms; repetition time, 3200 ms; slice thickness, 0.8 mm; number of slices, 208; voxel matrix, 320 × 300; and voxel size, 1.0 × 1.0 × 0.8 mm.

Imaging data were preprocessed using HCP pipelines, including volume segmentation and cortical surface reconstruction with FreeSurfer 6.0^43,44^. The T1w/T2w ratio was determined at multiple levels within the cortical ribbon. Specifically, the T1w/T2w ratio was calculated at 5% increments in cortical thickness from the gray-white boundary to the gray-cerebrospinal fluid boundary and averaged within 4 cortical ribbon levels as follows: 5%–25% (deep cortex), 30%–50% (lower-middle cortex), 55%–75% (upper-middle cortex), and 80%–100% (superficial cortex). The 4 resulting myelin maps were parcellated using the HCP multi-modal parcellation 1.0 atlas, resulting in 360 cortical regions with 4 T1w/T2w ratio estimates each.

### Statistics and reproducibility

Partial correlation coefficients, controlling for sex and age, were calculated to determine individual factors associated with worse ADI using SPSS version 28 (IBM Crop., Albany, NY, USA), with bootstrapping (5000 samples) (*n* = 92 for BMI and PSS; *n* = 81 for TFA intake). *P*-values <.05 were considered statistically significant.

Non-rotated partial least squares correlational (PLSC) analysis was applied to identify regions correlated with ADI according to cortical level (*n* = 92), using freely available code (http://www.rotman-baycrest.on.ca/pls)^45^. PLSC is a multivariate analytical technique that identifies weighted patterns of variables in two blocks of variables that maximally covary with each other^45,46^. In this study, one block comprised demographic variables (ADI, age, sex) and one block comprised parcellated T1w/T2w ratio values (at a specific cortical level). Weights were preset to identify brain regions sensitive to ADI but not sex or age. Reliability of identified regions was assessed using bootstrap estimation (5000 samples). The obtained bootstrap ratio is roughly equivalent to a z-score^45^; in the present study, regions with a bootstrap ratio >3.3 (corresponding to *p* < .001) were considered significant. For brain parcels with significance, T1w/T2w ratio data was extracted from those cortical levels with significance to calculate a weighted average (according to the size of the parcel) of the T1w/T2w ratio across all areas with a positive relationship with ADI (ADI-positive areas) and an average of all areas with a negative relationship with ADI (ADI-negative areas), for further analysis.

Structural equation modeling (SEM) was applied to investigate the mediation of relationships between ADI and significant findings from the PLSC analysis (*n* = 92). SEM was performed in R Studio using the lavaan package^47^. Input variables comprised ADI, BMI, PSS score, average T1w/T2w ratio in ADI-positive areas, and average T1w/T2w ratio in ADI-negative areas, as well as sex and age as control variables. Data were standardized prior to fitting the model. Missing values were estimated using the maximum likelihood (4 participants had missing PSS scores). Model fit was assessed using the chi-squared *p*-value, comparative fit index, and standardized root mean square residual. Model paths with a *p*-value <.05 were considered significant.

### Reporting summary

Further information on research design is available in the Nature Portfolio Reporting Summary linked to this article.

## Results

### Sample characteristics

Participant characteristics, including TFA intake, are summarized in Table 1. The mean age was 28.0 years (standard deviation, 10.3 years; range 18–58 years). In our sample, ADI was positively correlated with BMI (*r* = 0.27, *p* = .01) and PSS (*r* = 0.22, *p* = .04), but BMI and PSS were not correlated (*r* = 0.04, *p* = .73).

**Table 1:** Participant characteristics.

Variable	*N* (%) or Mean ± SD
Male	27 (29%)
Age (yrs)	28.0 ± 10.3
Hispanic (%)	37 (40%)
Race (%)	
Asian	34 (37%)
Black	5 (5%)
White	28 (30%)
Mixed	11 (12%)
Other	19 (21%)
Income (%)	
<20k	8 (9%)
20k–39k	14 (15%)
40k–59k	15 (16%)
60k–79k	17 (19%)
80k+	32 (35%)
Body mass index (kg/m^2^)	24.9 ± 4.7
Perceived Stress Scale score	15.05 ± 7.5
National ADI	11.4 ± 10.6
California ADI	4.0 ± 2.4
Trans-fatty acid intake	
Trans-hexadecenoic acid	0.03 ± 0.02
Trans-octadecenoic (elaidic) acid	1.38 ± 0.80
Trans-octadecadienoic (linolelaidic) acid	0.24 ± 0.14
Total	1.65 ± 0.93

Data are mean ± standard deviation, unless otherwise indicated.

*ADI* area deprivation index.

### Regions showing a relationship between ADI and T1w/T2w ratio according to cortical level

**Fig. 2:**
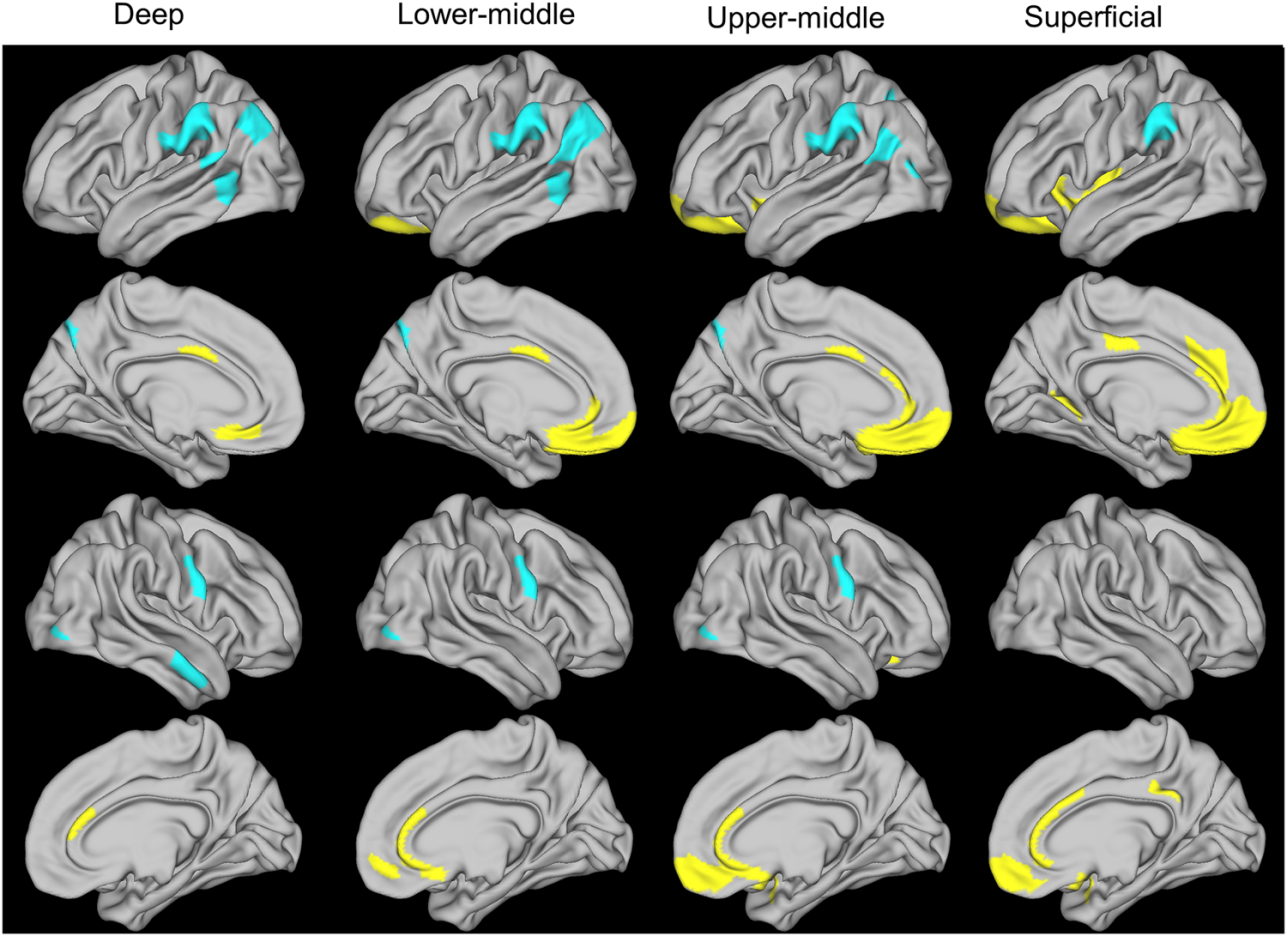
Relationship between worse ADI and cortical microstructure assessed as the T1w/T2w ratio at four cortical ribbon levels. The four cortical ribbon levels were as follows: deep, 5%–25% of cortical thickness from white-pial boundary; lower-middle, 30%–50%; upper-middle, 55%–75%, superficial, 80%–100%. Worse ADI was associated with increased T1w/T2w ratio (yellow) in medial prefrontal and cingulate regions, and decreased T1w/T2w ratio (blue) in supramarginal, middle temporal, and primary motor regions (*p* < .001) (*n* = 92). ADI area deprivation index, T1w/T2w, T1-weighted/T2-weighted.

The PLSC analysis revealed worse ADI as associated with increased T1w/T2w ratio in medial prefrontal and cingulate regions, mainly at middle/superficial cortical levels (referred to as ADI-positive areas); these regions are involved in reward-related processing, emotional regulation, and higher cognition^48–50^. Worse ADI was also associated with decreased T1w/T2w ratio in supramarginal, middle temporal, and primary motor regions in mainly middle/deep cortical levels (referred to as ADI-negative areas); these regions are components of the extended mirror system involved in social interaction (*p* < .001) (Fig. 2)^51–53^.

### Mediation of the relationship between ADI and T1w/T2w ratio

**Fig. 3:**
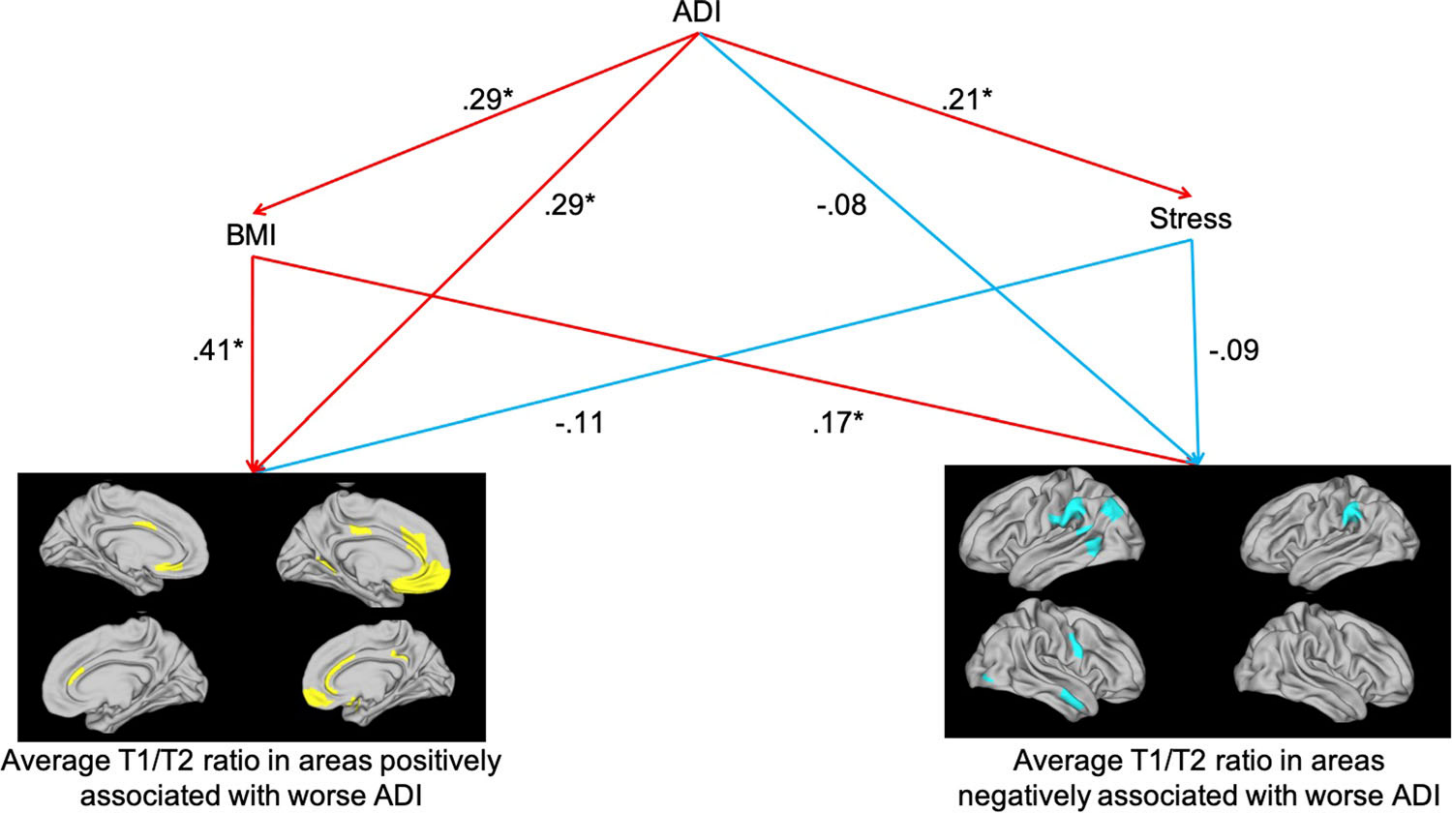
Structural equation modeling of the mediation of the relationships between worse ADI and cortical microstructure assessed as the T1w/T2w ratio. BMI partially mediated the relationship between ADI and the T1w/T2w ratio in areas positively associated with worse ADI (*n* = 92). Path coefficients reflect the direct effect of one variable on another variable, controlling for all other variables in the model (i.e. if X changes by 1 standard deviation, Y changes by b standard deviations, where b is the path coefficient). Red arrows indicate positive effects; blue arrows indicate negative effects; yellow clusters indicate ADI-positive areas; blue clusters indicate ADI-negative areas.*p < .05; ADI area deprivation index, BMI body mass index, T1w/T2w, T1-weighted/T2-weighted.

The final model is shown in Fig. 3; for simplicity, sex and age are not shown in the model, but were used as control variables. The model had a high chi-squared *p*-value (χ^2^(2) = 0.025, *p* = .99), comparative fit index of 1.0, and standardized root mean square residual of 0.002, indicating good model fit.

BMI had significant positive direct effects on both brain variables (ADI-positive areas: *Z* = 4.26, *p* < .001; ADI-positive areas: *Z* = 2.14, *p* = .03). Additionally, BMI partially mediated the relationship between ADI and the average T1w/T2w ratio in ADI-positive areas (indirect effect of ADI on ADI-positive areas via BMI: *Z* = 2.39, *p* = .02; accounting for 29% of the total effect of ADI on ADI-positive areas), but not ADI-negative areas (indirect effect of ADI on ADI-negative areas: *Z* = 1.71, *p* = 0.09).

Although stress (PSS score) had a negative direct effect on both brain variables, statistical significance was not reached (ADI-negative areas: *Z* = −1.10, *p* = 0.33; ADI-positive areas: *Z* = −1.02, *p* = 0.31). Stress also failed to reach significance as a mediator of the relationship between ADI and the average T1w/T2w ratio in these regions (indirect effect of ADI on ADI-positive areas via stress: *Z* = −0.99, *p* = .36; on ADI-negative areas: *Z* = −0.92, *p* = .32).

### Relationship between TFA intake and T1w/T2w ratio

Correlations between TFA intake and the T1w/T2w ratio are shown in Table 2. Significant positive correlations were observed between the average T1w/T2w ratio in ADI-positive areas and trans-octadecenoic (elaidic) acid (*r* = .29, *p* = .01) and total TFA intake (*r* = .28, *p* = .01). No significant correlations were observed for the average T1w/T2w ratio in ADI-negative areas.

**Table 2:** Correlations between TFA intake and the T1w/T2w ratio, controlling for sex and age.

	trans-hexadecenoic acid	trans-octadecenoic (elaidic) acid	trans-octadecadienoic (linolelaidic) acid	Total TFA
Areas positively associated with ADI	.10 (*p* = .38)	.29 (*p* = .01)	.18 (*p* = .12)	.28 (*p* = .01)
Areas negatively associated with ADI	.09 (*p* = .44)	.20 (*p* = .08)	.12 (*p* = .24)	.19 (*p* = .10)
BMI	.24 (*p* = .04)	.27 (*p* = .02)	.24 (*p* = .04)	.27 (*p* = .02)

*ADI* area deprivation index, *BMI* body mass index, *TFA* tans-fatty acid.

## Discussion

We examined the relationship between ADI and T1w/T2w ratio at different levels of the cortical ribbon, as well as potential mediation by factors associated with ADI, namely, BMI and stress. We found that ADI was associated with increased T1w/T2w ratio in medial prefrontal and cingulate cortices at middle/superficial levels (ADI-positive areas); these regions are involved in reward-related, emotion regulation, and higher cognitive processes^48–50^. This association was partially mediated by increased BMI. We also found that ADI was associated with decreased T1w/T2w ratio in supramarginal, middle temporal, and primary motor cortices at middle/deeper levels (i.e. ADI-negative areas); these regions are components of the mirror neuron system, involved in social interaction^51–53^. BMI did not appear to mediate this relationship. Further, perceived stress was associated with ADI but not the T1w/T2w ratio in evaluated areas. As elaborated below, these findings may provide insights as to the nature of affected information processing pathways under worse ADI.

In the present study, worse ADI was associated with increased T1w/T2w ratio in more superficial cortex in medial prefrontal and cingulate regions. These results are largely similar to a previous developmental study on the relationship between disadvantage due to low socioeconomic status (SES) and the T1w/T2w ratio. This previous study found that lower parental SES was associated with increased T1w/T2w ratio in frontal, temporal, medial parietal, and occipital regions in children and adolescents^54^. Thus, disadvantage due to ADI or individual SES may be associated with increased T1w/T2w ratio in overlapping regions. Further, superficial cortex receives top-down information from subcortical and cortical regions and is thought to enable flexible and state-dependent processing of feedforward sensory input arriving in deeper layers^32,55^. Thus, alterations in the microstructure of the superficial cortex could negatively influence the context and flexibility of information processing in affected regions, which in the present study, comprised the prefrontal and cingulate cortices. Given the involvement of these regions in reward-related processing, emotional regulation, and higher cognition, this interpretation is consistent with previous behavioral studies showing an impact of neighborhood disadvantage on these functions throughout the lifespan.

As the T1w/T2w ratio is sensitive to myelin content, one potential explanation for these results is that worse ADI is associated with increased intracortical myelination in ADI-positive areas. The maturation of intracortical myelination is protracted in humans, especially in prefrontal regions, peaking at 30–45 years of age depending on the brain region^30^. Accordingly, intracortical myelination within a normative range may be needed for optimal function, with both reduced and excessive myelination being problematic^56^. Consistent with the latter, animal studies have found that under conditions of excessive myelination (i.e., beyond axonal demand) mistargeting to cell bodies occurs readily^57^. Thus, our finding that worse ADI and increased BMI are associated with increased T1w/T2w ratio in more superficial cortex in medial prefrontal and cingulate regions may imply excessive and disorganized myelination in the upper layers of the cortex. However, studies using markers of intracortical myelination other than the T1w/T2w ratio have mainly found reductions associated with neighborhood or socioeconomic disadvantage. For example, a study using magnetization transfer as a myelin-sensitive marker, found that living in a disadvantaged neighborhood before the age of 12 years was associated with slower myelin growth in adolescents and young adults in sensorimotor, cingulate, and prefrontal cortices^17^. Another study using magnetization transfer found that SES in adulthood was associated with decreased entorhinal cortical myelination^58^.

Given our finding that increased T1w/T2w ratio in more superficial, but not deeper, cortical levels of prefrontal and cingulate regions was mediated by BMI, we entertained the possibility that the T1w/T2w ratio at the upper BMI range was affected by a fatty acid-rich cortical environment, with lipid droplet accumulation and lipid-laden astrocytes, due to blood-brain barrier disruption and increased transport of fatty acids under obese conditions^59–61^. In support of this, we found that total TFA intake, largely driven by trans-octadecenoic (elaidic) acid intake, was correlated with increased superficial cortical T1w/T2w ratio in ADI-positive areas. Although industrial TFAs, such as partially hydrogenated oil, have been banned in the United States (effective 2020) because of health concerns, the process of repeatedly cooking oil at high temperatures can still cause high levels of TFAs in fried fast foods^62^. A poor-quality diet, such as one high in fried fast foods, is thought to be one of the factors of worse ADI that contributes to obesity and worse health outcomes^3–5,63^. In particular, higher intake of TFAs, including elaidic acid, is associated with an increased risk of dementia^64,65^. Thus, we speculate that our results could alternatively suggest that a diet high in TFA under worse ADI creates a fatty acid-rich environment in superficial/middle cortical layers, disrupting information processing in affected regions. However, additional research, using other imaging modalities, is required to test this hypothesis.

We also found that worse ADI was associated with decreased T1w/T2w ratio in middle/deep cortical levels in supramarginal, middle temporal, and primary motor regions. These regions are components of the mirror neuron system, involved in understanding the actions of others and interpersonal coordination (e.g. imitation of another’s movements, cooperation between individuals)^51–53^. As middle/deep levels were involved, feed forward and feed-back processes and intercortical and subcortical-cortical communication in these regions may be affected with worse ADI^32^. A previous study found that higher perceived stress was associated with lower T1w/T2w ratio in the right supramarginal gyrus^18^. However, in the present study, perceived stress was not significantly associated with decreased T1w/T2w ratio in ADI-negative areas, which included the left, but not the right, supramarginal gyrus. Therefore, this relationship may be highly lateralized. Additionally, the PSS is an acute measure of stress (within 30 days). Chronic measures of stress may show more robust relationships; however, such parameters were not available in this dataset. Thus, further research with additional parameters is needed to elucidate the mediation of the association between worse ADI and decreased T1w/T2w ratio in these regions.

The present study has several limitations. First, the biological basis of the observed T1w/T2w alterations remains uncertain. Although the T1w/T2w ratio may be sensitive to myelin content, it has limited specificity and may also reflect neurite or synaptic density^27,28^. Further, we speculate that a fatty acid-rich cortical environment may contribute to increased T1w/T2w ratio. Additional studies using other acquisition protocols are needed to clarify the nature of the observed alterations. Second, we assessed current ADI at a single point in time; we did not have information regarding the length of residence or the amount of time since leaving the parents’ home, nor did we have historical data on ADI in younger ages. A previous study found that, although both childhood and adulthood SES showed correlations with intracortical myelination, childhood SES showed robust associations even after controlling for adult SES, suggesting a lasting imprint, which may also hold for neighborhood-level factors such as ADI. Further, the differential effects of the current vs the parents’ neighborhood environment may depend on the amount of time since leaving the parents’ home, limiting the generalizability of the results. Third, BMI has limitations in reflecting body adiposity, as it is influenced by excess muscle and does not provide information regarding the distribution of fat^66^. Fourth, a selection bias may exist as the race proportions in the present study differ from those in the 2020 Census^67^.

In conclusion, we found that worse ADI was associated with decreased T1w/T2w ratio in middle/deep cortical levels in supramarginal, middle temporal, and primary motor regions, potentially impacting intercortical and subcortical-cortical communication of the mirror neuron system, important for understanding the actions of others and cooperative behavior. ADI was also associated with increased T1w/T2w ratio in middle/superficial cortical levels in medial prefrontal and cingulate regions, which was partially mediated by increased BMI. Further, this increased T1w/T2w ratio was positively correlated with TFA intake. Thus, obesogenic features of neighborhood disadvantage may disrupt the flexibility of information processing involved in reward, emotion regulation, and cognition. These results provide additional information regarding the ramifications of living in a disadvantaged neighborhood on brain health. Further research on the mediating factors involved in the impact of ADI on the brain during development and adulthood is needed.

### Supplementary information

Reporting Summary

## Data Availability

The data analyzed for the current paper are not publicly available due to an ongoing funded study and, as per NIH policy, will be made available upon the completion of the larger prospective study. However, de-identified individual participant data (behavioral, brain) can be shared upon request and will be made available through our Center’s Pain Repository portal (https://www.painrepository.org/). To access the data, researchers fill out a user agreement, upon which access to the data will be made available through a secure password-protected portal.
