# Supplementary material for: Mediation of the association between disadvantaged neighborhoods and cortical microstructure by body mass index
Source: Commun Med (Lond). 2023 Sep 15;3:122. doi: 10.1038/s43856-023-00350-5 (PMC10504354; doi:10.1038/s43856-023-00350-5)
Supplement: Supplementary file 1 — Reporting Summary [file 43856_2023_350_MOESM1_ESM.pdf]

nature portfolio

Corresponding author(s): COMMSMED-23-0157B

Last updated by author(s): Aug 17, 2023

Reporting Summary

Nature Portfolio wishes to improve the reproducibility of the work that we publish. This form provides structure for consistency and transparency in reporting. For further information on Nature Portfolio policies, see our Editorial Policies and the Editorial Policy Checklist.

Statistics

For all statistical analyses, confirm that the following items are present in the figure legend, table legend, main text, or Methods section.

n/a	Confirmed	
☐	☒	The exact sample size (*n*) for each experimental group/condition, given as a discrete number and unit of measurement
☐	☒	A statement on whether measurements were taken from distinct samples or whether the same sample was measured repeatedly
☐	☒	The statistical test(s) used AND whether they are one- or two-sided<br>*Only common tests should be described solely by name; describe more complex techniques in the Methods section.*
☐	☒	A description of all covariates tested
☐	☒	A description of any assumptions or corrections, such as tests of normality and adjustment for multiple comparisons
☐	☒	A full description of the statistical parameters including central tendency (e.g. means) or other basic estimates (e.g. regression coefficient) AND variation (e.g. standard deviation) or associated estimates of uncertainty (e.g. confidence intervals)
☐	☒	For null hypothesis testing, the test statistic (e.g. *F*, *t*, *r*) with confidence intervals, effect sizes, degrees of freedom and *P* value noted<br>*Give P values as exact values whenever suitable.*
☒	☐	For Bayesian analysis, information on the choice of priors and Markov chain Monte Carlo settings
☒	☐	For hierarchical and complex designs, identification of the appropriate level for tests and full reporting of outcomes
☐	☒	Estimates of effect sizes (e.g. Cohen's *d*, Pearson's *r*), indicating how they were calculated

Our web collection on statistics for biologists contains articles on many of the points above.

Software and code

Policy information about availability of computer code

Data collection	Neighborhood Atlas, open database of the area deprivation index by census tract
Data analysis	PLSCMD, open source tool for partial least squares analysis; lavaan package for R Studio, open source tool for structural equation modeling; SPSS for descriptive data and partial correlation analysis

For manuscripts utilizing custom algorithms or software that are central to the research but not yet described in published literature, software must be made available to editors and reviewers. We strongly encourage code deposition in a community repository (e.g. GitHub). See the Nature Portfolio guidelines for submitting code & software for further information.

Data

Policy information about availability of data

All manuscripts must include a data availability statement. This statement should provide the following information, where applicable:

- Accession codes, unique identifiers, or web links for publicly available datasets
- A description of any restrictions on data availability
- For clinical datasets or third party data, please ensure that the statement adheres to our policy

The datasets generated and/or analyzed during the current study are not publicly available due to an ongoing funded study and, as per NIH policy, will be made available upon the completion of the larger prospective study. However, deidentified individual data (behavioral, brain) can be shared upon request and will be

made available through our Center's Pain Repository portal (https://www.painrepository.org). To access the data, researchers fill out a user agreement, uplon which access to the data will be made available through a secure password-protected portal.

Human research participants

Policy information about studies involving human research participants and Sex and Gender in Research.

Reporting on sex and gender	There were 27 men and 65 women. Due to the relatively small number of men, we were not sufficiently powered to evaluate sex differences in this study.
Population characteristics	Participants comprised healthy adults with an average ave of 28.0 (standard deviation: 10.3) years.
Recruitment	Participants were recruited by flyers posted on the University of California, Los Angeles (UCLA) campus and doctor offices and handed out at community/church events in the Los Angeles area, as well as by mass emails to the UCLA community and listings on social media and clinicaltrials.gov (NCT05120908).
Ethics oversight	Data were obtained from studies approved by the Office of Protection for Research Subjects at the University of California, Los Angeles (Nos. 16-000281, 20-000549, 20-00515, 20-002326). IRB approval specific to the present study was not required as de-identified data under approval were used.

Note that full information on the approval of the study protocol must also be provided in the manuscript.

Field-specific reporting

Please select the one below that is the best fit for your research. If you are not sure, read the appropriate sections before making your selection.

☒ Life sciences ☐ Behavioural & social sciences ☐ Ecological, evolutionary & environmental sciences

For a reference copy of the document with all sections, see nature.com/documents/nr-reporting-summary-flat.pdf

Life sciences study design

All studies must disclose on these points even when the disclosure is negative.

Sample size	No sample-size calculation was performed. The sample was collected within a specified period of time and is within the range of similar nerroimaging studies.
Data exclusions	Individuals with major neurological condition, current or past psychiatric illness, vascular disease, weight loss/abdominal surgery, substance use disorder, use of medications that interfere with the central nervous system, pregnant or breastfeeding, strenuous exercise regimen (>8 h/week of continuous exercise), weight >400 pounds, or metal implants were excluded. In addition, enrolled participants with poor quality images were excluded; however, none of the enrolled participants were deemed to have poor quality images
Replication	No replication has been performed.
Randomization	There were no experimental groups
Blinding	There were no experimental groups.

Reporting for specific materials, systems and methods

We require information from authors about some types of materials, experimental systems and methods used in many studies. Here, indicate whether each material, system or method listed is relevant to your study. If you are not sure if a list item applies to your research, read the appropriate section before selecting a response.

Materials & experimental systems

n/a	Involved in the study	
☒	☐	Antibodies
☒	☐	Eukaryotic cell lines
☒	☐	Palaeontology and archaeology
☒	☐	Animals and other organisms
☒	☐	Clinical data
☒	☐	Dual use research of concern

Methods

n/a	Involved in the study	
☒	☐	ChIP-seq
☒	☐	Flow cytometry
☐	☒	MRI-based neuroimaging

Magnetic resonance imaging

Experimental design

Design type	Structural MRI
Design specifications	N/A
Behavioral performance measures	N/A

Acquisition

Imaging type(s)	Structural MRI
Field strength	3T
Sequence & imaging parameters	T1w: TE, 1.81 ms; TR, 2500 ms; slice thickness, 0.8 mm; number of slices 208; voxel matrix 320x300; voxel size 1.0x1.0x0.8 mm<br>T2w:TE, 564 ms; TR, 3200 ms; slice thickness, 0.8 mm; number of slices 208; voxel matrix 320x300; voxel size 1.0x1.0x0.8 mm
Area of acquisition	Whole brain

Diffusion MRI ☐ Used ☒ Not used

Preprocessing

Preprocessing software	Freesurfer 6.0; Human Connectome Project pipeline 4.3
Normalization	Nonlinear transformation to MNI template
Normalization template	MNI template
Noise and artifact removal	Spin echo fieldmaps collected in AP and PA directions were used for artifact removal
Volume censoring	N/A

Statistical modeling & inference

Model type and settings	Partial least squares correlational analysis (5000 bootstraps) and structural equation modeling (missing values estimated using the maximum likelihood).
Effect(s) tested	Correlations between area deprivation index and cortical microstructure; mediation by body mass index and/or chronic stress

Specify type of analysis: ☐ Whole brain ☐ ROI-based ☒ Both

Anatomical location(s)	partial least squares on parcellated regions of whole brain, followed by structural equation modeling with significant regions
Statistic type for inference (See Eklund et al. 2016)	Parcellated regions
Correction	Bootstrap sampling

Models & analysis

n/a	Involved in the study
☒	☐ Functional and/or effective connectivity
☒	☐ Graph analysis
☒	☐ Multivariate modeling or predictive analysis
